# Cold Agglutinins and Cryoglobulins Associate With Clinical and Laboratory Parameters of Cold Urticaria

**DOI:** 10.3389/fimmu.2021.665491

**Published:** 2021-04-29

**Authors:** Mojca Bizjak, Mitja Košnik, Dorothea Terhorst-Molawi, Dejan Dinevski, Marcus Maurer

**Affiliations:** ^1^ Urticaria Center of Reference and Excellence (UCARE), University Clinic of Respiratory and Allergic Diseases Golnik, Golnik, Slovenia; ^2^ Urticaria Center of Reference and Excellence (UCARE), Dermatological Allergology, Allergie-Centrum-Charité, Department of Dermatology and Allergy, Charité - Universitätsmedizin Berlin, Berlin, Germany; ^3^ Faculty of Medicine, University of Maribor, Maribor, Slovenia

**Keywords:** cold agglutinin, cryoglobulin, cold urticaria, mast cell, degranulation, clinical parameters, laboratory parameters, cold triggers

## Abstract

Mast cell-activating signals in cold urticaria are not yet well defined and are likely to be heterogeneous. Cold agglutinins and cryoglobulins have been described as factors possibly associated with cold urticaria, but their relevance has not been explained. We performed a single-center prospective cohort study of 35 cold urticaria patients. Cold agglutinin and cryoglobulin test results, demographics, detailed history data, cold stimulation test results, complete blood count values, C-reactive protein, total immunoglobulin E levels, and basal serum tryptase levels were analyzed. Forty six percent (*n* = 16) of 35 tested patients had a positive cold agglutinin test and 27% (*n* = 9) of 33 tested patients had a positive cryoglobulin test. Cold agglutinin positive patients, when compared to cold agglutinin negative ones, were mainly female (*P* = 0.030). No gender-association was found for cryoglobulins. A positive cold agglutinin test, but not a positive cryoglobulin test, was associated with a higher rate of reactions triggered by cold ambient air (*P* = 0.009) or immersion in cold water (*P* = 0.041), and aggravated by increased summer humidity (*P* = 0.007). Additionally, patients with a positive cold agglutinin test had a higher frequency of angioedema triggered by ingestion of cold foods or drinks (*P* = 0.043), and lower disease control based on Urticaria Control Test (*P* = 0.023). Cold agglutinin levels correlated with erythrocyte counts (r = −0.372, *P* = 0.028) and monocyte counts (r = −0.425, *P* = 0.011). Cryoglobulin concentrations correlated with basal serum tryptase levels (r = 0.733, *P* = 0.025) and cold urticaria duration (r = 0.683, *P* = 0.042). Results of our study suggest that cold agglutinins and cryoglobulins, in a subpopulation of cold urticaria patients, are linked to the course and possibly the pathogenesis of their disease.

## Introduction

Cold urticaria (ColdU) is a type of chronic inducible urticaria (CIndU) characterized by the occurrence of wheals and/or angioedema in response to cooling (1, 2). It is classified into: (a) cold contact urticaria, which is characterized by whealing in response to local cold stimulation tests (CSTs) on the forearm with an ice cube and/or Temp*Test* ^®^ technology, and (b) atypical ColdU, in which other provocation methods are needed to produce whealing (e.g., general body cooling) or local CSTs produce atypical responses. Cold-induced anaphylaxis may also occur (1). ColdU is confirmed by provocation testing, and disease activity is measured by trigger threshold(s) (3–5). According to the EAACI/GA^2^LEN/EDF/WAO urticaria guideline, laboratory tests in ColdU are recommended only as an extended diagnostic approach to rule out other diseases, especially infections (3). In most patients, no underlying causes can be found (6).

ColdU is a mast cell-driven disease, where activating signals cause release of histamine from dermal mast cells (7–9). Histamine release in ColdU coincides with the onset of pruritus and swelling (10). The mast cell-activating signals in ColdU have not yet been well defined and are likely to be heterogeneous (1, 3). Autoantibodies are held to play a role in some patients with ColdU (1), and passive transfer studies found that localized ColdU can be induced in healthy human subjects by injecting them intradermally with serum of some ColdU patients (11). Further studies found that the serum factors that initiated ColdU depended on immunoglobulin E (IgE) (10, 12, 13), and rarely on immunoglobulin M (IgM) (14). A cold-inducible antigen, however, has not yet been identified (1).

Cold agglutinins (CAs) are cold-reactive antibodies that are able to agglutinate erythrocytes (15). Silpa-archa et al. found CAs in 40% of 20 studied ColdU subjects and reported “mainly low titers” (16). CAs were also described in a few ColdU case reports (17–19). The majority of CAs are IgM proteins (20), but few cases of immunoglobulin A (IgA) or immunoglobulin G (IgG) CAs have also been described (15, 21–23). The ability of CAs to agglutinate erythrocytes after binding to their cell surface can be attributed to the pentameric structure and large molecular size of IgM. Most CAs are directed against the Ii-blood group system of carbohydrate antigens (15). CAs are assessed semi-quantitatively by the CA titer, usually first measured at 4°C and defined as the inverse of the maximum serum dilution at which agglutination of donor erythrocytes is seen *in vitro*. The thermal amplitude is defined as the highest temperature at which the CA will react with its antigen (24). Interestingly, studies in hematology have shown that the pathogenicity of CAs depends more on the thermal amplitude, which can approach 37°C, than on the CA titer (15, 23, 25).

CAs have been mostly studied for their pathogenic role in cold-antibody autoimmune hemolytic anemias: (a) CA disease, a well-defined clonal B-cell lymphoproliferative disorder, and (b) CA syndrome, most frequently caused by *Mycoplasma pneumoniae*, Epstein-Barr virus, or aggressive lymphoma. Nearly all patients with CA disease have a CA titer ≥64 (**Table 1**) (15). At decreased ambient temperatures, cooling of peripheral skin sites (e.g., fingers, toes, ears, tip of the nose) allows binding of the CAs to erythrocytes in acral capillaries and their agglutination with subsequent possible cold-induced ischemic manifestations (e.g., acrocyanosis, Raynaud’s phenomenon, rarely gangrene) (15, 21, 23). CA binding to erythrocytes activates the classical complement pathway and may cause hemolysis (15, 28). ColdU is a rare feature of CA disease (29). CAs are also found in a proportion of the adult population without any hemolysis or clinical symptoms. Such CAs are present in low titers (mostly <64), have low thermal amplitude, and are polyclonal (**Table 1**) (15). In a study of 14900 patients screened prior to a cardiopulmonary bypass procedure, 0.3% tested positive for CAs (30). Non-pathogenic CAs may be remnants of a primitive vertebrate immune system (15) and the product of either random rearrangement of the immunoglobulin gene segments in the bone marrow and/or produced as a result of molecular mimicry with structures on the surface of infectious agents (31).

**Table 1:** Cold agglutinin and cryoglobulin properties.

CA properties (15)	
CA disease	Anti-I (rarely anti-Pr or anti-IH) CA titer usually ≥64 at 4°C
CA syndrome	Anti-I or anti-i IgM or IgG
Normally occurring CAs	CA titer usually <64 at 4°C Low thermal amplitude Polyclonal
**CG properties** (26, 27)	
Single-component CGs	One Ig isotype (IgM or IgG, rarely IgA) Monoclonal Ig CG concentration often >2000 mg/L
Mixed CGs	Two different Ig isotypes (usually IgM and IgG) (a) Monoclonal Ig + polyclonal Ig or (b) polyclonal Ig
Normally occurring CGs	CG concentration <20 mg/L Mixed, polyclonal Ig

CA, cold agglutinin; CG, cryoglobulin; Ig, immunoglobulin.

Cryoglobulins (CGs) are antibodies that precipitate *in vitro* at temperatures below 37°C and dissolve after rewarming (26, 32, 33). Their precipitation *in vivo* can cause vasculitis of small and medium-sized vessels in the skin, joints, nerves and kidneys (26), and consequently signs and symptoms such as intermittent purpura, livedo, leg ulcers, acrocyanosis, Raynaud’s phenomenon, arthralgias, and symptoms due to peripheral neuropathy (26, 34, 35). CGs have been detected in up to 10% of ColdU patients studied in 4 case series with 71-208 enrolled patients (36–39). Additionally, a few case reports of CG-positive ColdU patients have been published (40–43). CGs are divided into: (a) single-component monoclonal CGs, which contain only one immunoglobulin isotype (IgG or IgM, rarely IgA), and (b) mixed CGs, which are immune complexes composed of two different immunoglobulin isotypes, most commonly IgM and IgG (26, 27). Mixed CGs are often found secondary to chronic (viral, bacterial or parasitic) infections (26). In studies performed by Sidana et al. (34), Dammacco et al. (35), and Costanzi and Coltman (43), ColdU was found in 2−4% of patients with CGs. Healthy individuals may have CGs at low concentrations (26, 27, 44). CGs are different from CAs (20, 45), although an IgM cryoprotein with both CA- and CG-properties has been described (46).

So far nothing is known about the impact of CAs and CGs on ColdU features on the molecular or clinical level, and no evaluation strategies have been proposed for CA-positive and CG-positive ColdU patients. In practice, referrals of these patients to hematologists and rheumatologists does not help guide further evaluation and treatment of ColdU. Consequently, we designed a comprehensive study with the aim to overcome these knowledge gaps. Detailed analysis of cold triggers has not yet been undertaken either and the effects of different relevant cold triggers on the clinical presentation of ColdU are still incompletely understood (1).

## Materials and Methods

### Patients

Thirty five consecutive adult ColdU patients, who were referred to the Urticaria Center of Reference and Excellence (UCARE) at the University Clinic Golnik (47), were recruited. Their age ranged from 18 to 73 years (mean 41.4, SD ± 13.4). There were 66% (*n* = 23) women and 34% (*n* = 12) men (**Table 2**). All (*n* = 35) patients had ColdU based on their history and local CSTs were performed. Routine physical examination was also done. The study spanned through all seasons (from May 1, 2019 to March 31, 2020) and it was approved by the National Medical Ethics Committee of the Republic of Slovenia. Patients gave written informed consent.

**Table 2:** Characteristics of patients with a positive *vs*. negative cold agglutinin test.

Parameter	CA test			
	Total (*N* = 35)	Negative (*N* = 19)	Positive (*N* = 16)	*P* value (positive *vs*. negative)
Female sex	23 (66)	**9 (47)**	**14 (88)**	0.030*
Triggers				
Cold ambient air	28 (80)	**12 (63)**	**16 (100)**	0.009*
Immersion in <25°C water	19 (54)	**7 (37)**	**12 (75)**	0.041*
Higher summer humidity levels	15 (43)	**4 (21)**	**11 (69)**	0.007*
Ingestion of cold foods/drinks	13 (37)	**4 (21)**	**9 (56)**	0.043*
UCT score	12 (7−14)	**13 (9−16)**	**11 (7**−**12)**	**0.023** ^‡^
CST results				
Positive ice cube test	21 (60)	11 (58)	10 (63)	1.000
Positive Temp*Test* **^®^** result	12 (34)	5 (26)	7 (44)	0.311
CSTT (s)	300 (30−300); *N* = 19	300 (98−300); *N* = 10	120 (30−300); *N* = 9	0.150
CTT (°C)	19 (15−25); *N* = 12	17 (16−25); *N* = 5	21 (14−24); *N* = 7	0.622
Laboratory findings				
Erythrocyte count (10^12^/L)	4.7 ± 0.4	**4.9 ± 0.4**	**4.5 ± 0.3**	**0.005** ^†^
Hemoglobin concentration (g/L)	142.6 ± 10.4	**148.4 ± 8.7**	**135.8 ± 7.8**	**<0.001** ^†^
Hematocrit level (%)	41.7 ± 3.3	**43.2 ± 3.4**	**40.0 ± 2.5**	**0.004** ^†^
Thrombocyte count (10^9^/L)	265.9 ± 62.3	**246.4 ± 52.3**	**289.0 ± 66.9**	**0.042** ^†^
Monocyte count (10^9^/L)	0.5 ± 0.2	**0.6 ± 0.2**	**0.4 ± 0.2**	**0.002** ^†^
Leukocyte count (10^9^/L)	6.9 ± 1.7	7.3 ± 1.7	6.3 ± 1.5	0.070
Neutrophil count (10^9^/L)	4.2 ± 1.3	4.5 ± 1.3	3.8 ± 1.2	0.121
Lymphocyte count (10^9^/L)	1.9 ± 0.5	2.0 ± 0.5	1.9 ± 0.5	0.630
Eosinophil count (10^9^/L)	0.2 ± 0.3	0.1 ± 0.1	0.1 ± 0.1	0.324
Basophil count (10^9^/L)	0.04 ± 0.03; *N* = 34	0.04 ± 0.03; *N* = 19	0.04 ± 0.03; *N* = 15	0.620
CRP (mg/L)	1.7 (0.5−3.4)	1.2 (0.5−3.2)	2.1 (0.7−4.5)	0.320
Basal serum tryptase (ng/mL)	5.5 (3.9−7.6); *N* = 34	6.1 (4.3−8.7); *N* = 18	5.3 (3.7−6.0)	0.352
Total IgE (IU/mL)	99 (36−207); *N* = 31	82 (24−257); *N* = 15	113 (50−201)	0.782
Mean daily Temp. on the day when blood was drawn	12.1 ± 7.3	**9.3 ± 6.6**	**12.1 ± 7.3**	**0.008** ^†^

Data are given as no. (%), mean ± SD, and median (IQR). If data was not obtained in all patients, patient numbers are displayed as “N” next to results. Statistical significance of differences between patient groups was calculated by *Fisher’s Exact test, ^‡^Mann-Whitney U test, and ^†^Independent-samples T test. Statistically significant P values are in bold. ColdU, cold urticaria; CA, cold agglutinin; CRP, C-reactive protein; CST, cold stimulation test; CSTT, critical stimulation time threshold; CTT, critical temperature threshold; IQR, interquartile range; N, number of patients; s, second; SD, standard deviation; UCT, Urticaria Control Test.

### Patient History

Detailed history data focusing on numerous clinical parameters were obtained in all (*n* = 35) patients. The age at ColdU onset ranged from 9 to 60 years (mean 33.5, SD ± 12.5), and 9% of patients had a pediatric-onset ColdU (≤18 years). The duration of ColdU ranged from 2 to 384 months (median 60, IQR 15−156). The following frequencies of predefined cold-induced reactions (CRs) were reported: itch in 94% (*n* = 33), wheals in 100% (*n* = 35), angioedema in 37% (*n* = 13), gastrointestinal symptoms in 17% (*n* = 6), respiratory symptoms in 40% (*n* = 14), and symptoms of reduced blood pressure in 29% (*n* = 10). Eleven percent (*n* = 4) of patients reported cold-induced gastrointestinal and respiratory symptoms, 3% (*n* = 1) experienced gastrointestinal symptoms and symptoms of reduced blood pressure, and 26% (*n* = 9) of patients had respiratory and hypotensive symptoms. Maximal duration of wheals ranged from 5 to 720 minutes (median 60, IQR 20−120), and the maximal duration of angioedema ranged from 20 to 720 minutes (median 60, IQR 30−120).

A special emphasis in history taking was given to the relevance of 6 predefined cold triggers and 3 predefined aggravating factors. Triggers of at least one CR were the following: cold ambient air in 80% (*n* = 28), immersion in <25°C water in 54% (*n* = 19), immersion in ≥25°C water in 20% (*n* = 7), localized contact with cold liquids in 60% (*n* = 21), contact with cold surfaces in 54% (*n* = 19), and ingestion of cold foods or beverages in 51% (*n* = 18) of patients. Factors that aggravated at least one CR were: wind in 63% (*n* = 22), increased humidity levels in the summer (e.g., morning humidity, rain, walking barefoot on grass) in 43% (*n* = 15), and increased non-summer humidity levels (e.g. rain in cold seasons) in 43% (*n* = 15) of patients.

Treatment efficacy with daily second-generation H_1_-antihistamines in up to four times the licensed dose was graded as good in 23% (*n* = 8), moderate in 37% (*n* = 13), low in 9% (*n* = 3), no efficacy in 3% (*n* = 1), and unknown (not taken on a daily basis) in 29% (*n* = 10) of patients. Omalizumab treatment was required in 17% (*n* = 6) of patients. Nine percent (*n* = 3) of patients had at least one of the following cold-induced extracutaneous symptoms: fever, malaise, headache, eye redness, muscle pain, joint pain, or bone pain. Seventeen percent (*n* = 6) of patients also had chronic spontaneous urticaria, 6% (*n* = 2) symptomatic dermographism, 9% (*n* = 3) cholinergic urticaria, and 3% (*n* = 1) delayed pressure urticaria, but ColdU was a dominant disease in all these patients. Provocation tests for other CIndU types was done only in case of a suggestive history. Further history revealed the following comorbidities: at least one atopic disease (asthma, allergic rhinitis, allergic conjunctivitis, atopic dermatitis) in 40% (*n* = 14), asthma in 34% (*n* = 12), thyroid disease in 17% (*n* = 6), Raynaud’s phenomenon in 9% (*n* = 3), malignancy in 3% (*n* = 1), and autoimmune connective tissue disease in 3% (*n* = 1) of patients. Six percent (*n* = 2) of patients had a family history of ColdU.

### Patient Questionnaires

All (*n* = 35) patients completed the Urticaria Control Test (UCT) questionnaire containing four questions to determine the level of disease control. UCT scores ranged from 3 to 16 (median 12, IQR 7−14) (**Table 2**). A score ≤11 indicates insufficient disease control, whereas a score ≥12 suggests adequate disease control (3, 4, 48).

### Cold Stimulation Tests

Local CSTs on the volar forearm with an ice cube and a validated instrument Temp*Test* ^®^ were done in all (*n* = 35) patients. H_1_-antihistamines and systemic glucocorticoids were discontinued at least 3 and 7 days prior to CSTs, respectively (3–5). A five-minute stimulation with a melting ice cube in a non-latex glove was done first. Skin response was assessed 10 minutes after the end of stimulation. If a positive reaction was observed (i.e., whealing on the cold stimulated area on the forearm), shorter stimulation times (30 seconds, 1 minute, 2 minutes, 3 minutes, 4 minutes) were used to determine the shortest time needed for whealing (i.e., critical stimulation time threshold, CSTT). A 5-minute stimulation with the Temp*Test^®^* and reading 10 minutes after the end of stimulation was used to determine the highest temperature that can induce whealing in a specific patient (i.e., critical temperature threshold, CTT).

### Cold Agglutinin Test

Blood samples for CA analysis need to be kept at 37–38°C from sampling to separation of serum to avoid false low values and low sensitivity (49). To avoid this, all (*n* = 35) patients were referred to a dislocated Laboratory of Blood Transfusion Centre of Slovenia, where venous blood samples were collected and screened for the presence of CAs. The patients’ serum was mixed with suspensions (0.9% sodium chloride) of group O and RhD positive erythrocytes obtained from 5 random blood donors, lightly shaken, incubated at 4°C for 2 hours, and scored for macroscopic agglutination. The test was reported positive if blood clot was readily visible. Blood donor erythrocytes that produced the strongest agglutination were used for further analyses with dilutions of patient’s sera. The highest dilution able to agglutinate suspensions of erythrocytes at 4°C was recorded as the CA titer. Mean daily temperature on the day when blood for CA screening was drawn was obtained from the national weather service website (**Supplementary Table 1**).

### Cryoglobulin Test

Screening for the presence of CGs was performed in 94% (*n* = 33) of patients at the Laboratory for Immunology of University Medical Centre Ljubljana. Patients were referred to this dislocated center to prevent improper sample collection and transport since it is crucial to maintain blood samples at 37 ± 2°C from the collection to the laboratory analysis (26). Blood samples were allowed to clot at 37°C for 2 hours and then centrifugated at 37°C. Sera were decanted in conical bottom test tubes and placed for 7 days at 4°C. After this incubation, cryoprecipitate was isolated by cold centrifugation and purified using 3 washes with centrifugation in cold phosphate-buffered saline to remove other serum proteins. Following the last wash, phosphate-buffered saline together with reagent for facilitating immune complex dissociation was added to the samples. These samples were then placed at 37°C for up to 1 hour to dissolve the precipitate for further analyses. The detected cryoprecipitate was confirmed by quantification with a spectrophotometer at 720 nm. A value below 100 mg/L was reported as a negative result, whereas values of 100 mg/L or higher were reported as precise CG concentrations. If the first stage was positive, the second qualitative stage was also done. It involved radial immunodiffusion for characterization of CG isotypes (IgG, IgM, IgA) based on the presence or absence of a precipitation ring (**Supplementary Table 2**). Mean daily temperature on the day when blood for CG screening was drawn was also obtained (**Supplementary Table 3**).

### Other Laboratory Tests

Blood tests for most patients also included complete blood count with differential analysis, C-reactive protein (CRP), basal serum tryptase (ImmunoCAP 100 Thermo Fisher Scientific, Uppsala, Sweden), and total IgE (**Table 2**).

### Statistical Analyses

Data from completed paper survey documents were transferred to an electronic databank and IBM SPSS Statistics version 27 was used for analysis. Numerical variables were first assessed for normality distribution using visualization and Shapiro-Wilk test of normality. Descriptive measures included proportions, median with the first and third quartile range, and mean with standard deviation. Categorical variables were assessed using the Fisher’s Exact test. Numerical continuous variables with normal distribution were analyzed using parametric Independent-samples T test. Numerical variables that didn’t meet parametric assumptions as well as ordinal variables were analyzed using Mann-Whitney U test. Relationships between continuous variables were assessed with Spearman’s correlation. Spearman correlation coefficient was interpreted as follows: 0.20−0.39 weak, 0.40−0.59 moderate, and 0.60−0.79 strong. Tables and figures were employed to summarize the data.

## Results

### Cold Stimulation Test Results

Sixty percent (*n* = 21) of patients had a positive ice cube test and 34% (*n* = 12) of them had a positive Temp*Test^®^* result (**Table 2**). All patients with negative skin tests reported wheals on cold exposure. All Temp*Test* ^®^-positive patients also had a positive ice cube test, but only 57% (*n* = 12) of ice cube positive patients had a positive Temp*Test* ^®^ (*P* = 0.001). CSTT scores ranged from 30 to 300 seconds (median 300, IQR 30−300) and CTT scores ranged from 13 to 25°C (median 19, IQR 15─25) (**Table 2**). Cold contact urticaria (i.e., whealing on area on the forearm stimulated by an ice cube and/or Temp*Test* ^®^) was diagnosed in 63% (*n* = 22) of patients. In one of these patients, local CSTs were negative at the point of enrollment, but the diagnosis was established based on past positive local CSTs. The rest of the patients (*n* = 13) had atypical forms of ColdU(1): 29% (*n* = 10) had systemic atypical ColdU, one had localized ColdU, and 6% (*n* = 2) had localized cold reflex urticaria.

### Our Patient Cohort Has a High Frequency of CA-Positive and CG-Positive Patients

Almost half of our patients, 16 of 35 (46%), tested positive for CAs (**Table 2**), and their CA titers ranged from 1 to 16 (**Supplementary Table 1**). CGs were detectable in 27% (*n* = 9) of 33 tested patients (**Supplementary Table 2** and **Supplementary Table 3**). Two patients had single-component CGs of the IgG isotype, and 7 patients had mixed CGs. The maximum CG concentration was 189 mg/L (mean 152.0, SD ± 29.5) (**Supplementary Table 2**). Six percent (*n* = 2) of patients had a positive CA and CG test. No patients had a CA disease or a clinically manifested cryoglobulinemia.

### CAs, but Not CGs, Are Linked to Female Gender

**Figure 1:**
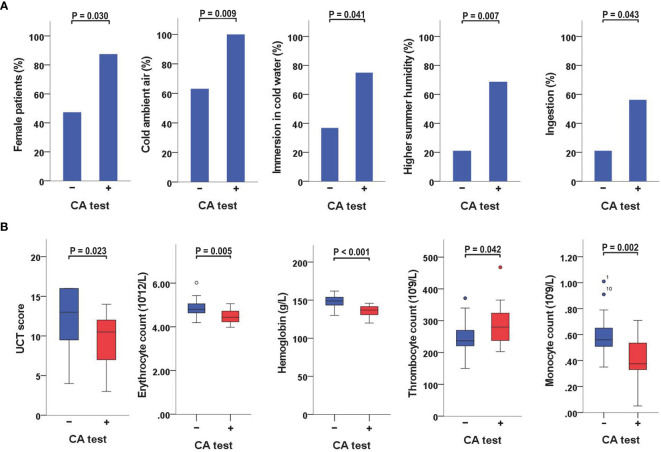
Variables significantly linked to a positive CA-test **(A)** When CA-positive and CA-negative patients were compared, the former were more often female and had a higher rate of reactions triggered by cold ambient air or immersion in cold water, a higher rate of reactions aggravated by increased humidity levels in the summer, and a higher frequency of angioedema triggered by ingestion of cold foods or drinks. **(B)** When CA-positive and CA-negative patients were compared, the former also had lower UCT scores, lower erythrocyte counts, lower hemoglobin concentrations, higher thrombocyte counts, and lower monocyte counts. CA, cold agglutinin; UCT, Urticaria Control Test.

CA-positive patients were more often female than CA-negative patients (88% *vs*. 47%, *P* = 0.030) (**Table 2** and **Figure 1A**). No gender-association was found for CGs (**Supplementary Table 3**).

### The Presence of CAs, but Not CGs, Influences the Patients’ Real-Life Reactivity to Specific Cold Triggers

When CA-positive and CA-negative patients were compared, the former had a higher rate of reactions triggered by cold ambient air (100% *vs*. 63%, *P* = 0.009), immersion in <25°C water (75% *vs*. 37%, *P* = 0.041), or aggravated by increased humidity levels in the summer (69% *vs*. 21%, *P* = 0.007). They also had a higher rate of angioedema triggered by ingestion of cold foods or drinks (56% *vs*. 21%, *P* = 0.043) (**Table 2** and **Figure 1A**). No significant associations with specific cold triggers were found for CGs (**Supplementary Table 3**).

### CAs, but Not CGs, Are Linked to Poor Disease Control

CA-positive patients had significantly lower UCT scores than CA-negative patients (median 11, IQR 7−12 *vs*. median 13, IQR 9−16; *P* = 0.023) (**Table 2** and **Supplementary Table 1**). Furthermore, CA titers weakly correlated with UCT scores (r = −0.359, *P* = 0.034) (**Table 3** and **Supplementary Table 1**). No significant differences in UCT scores were found between CG-positive and CG-negative patients (**Supplementary Table 3**).

**Table 3:** Correlations of cold agglutinin titers and cryoglobulin concentrations with patient characteristics.

Patient characteristic	CA titer *N* = 35		CG concentration *N* = 9	
	r	*P* value	r	*P* value
UCT score	−**0.359**	**0.034**	−0.059	0.880
Erythrocyte count	−**0.372**	**0.028**	0.059	0.881
Hemoglobin concentration	−**0.557**	**0.001**	0.000	1.000
Hematocrit level	−**0.397**	**0.018**	0.200	0.606
Monocyte count	−**0.425**	**0.011**	−0.017	0.966
Basal serum tryptase	−0.105	0.554	**0.733**	**0.025**
Mean daily Temp. on the day when blood was drawn	**0.456**	**0.006**	−0.663	0.073
ColdU duration	0.158	0.365	**0.683**	**0.042**

Statistically significant P values are in bold****. CA, cold agglutinin; CG, cryoglobulin; ColdU, cold urticaria; N, number of patients; r, Spearman correlation coefficient; Temp., temperature; UCT, Urticaria Control Test.

### A Positive CA test, but Not a Positive CG test, Was Associated With Specific Complete Blood Count Parameters

A positive CA test was linked to lower erythrocyte counts (*P* = 0.005) and lower hemoglobin concentrations (*P <*0.001) (**Table 2**; **Figure 1B**; **Supplementary Table 1**). CA titers correlated weakly with erythrocyte counts (r = −0.372, *P* = 0.028), moderately with hemoglobin concentrations (r = −0.557, *P* = 0.001), and moderately with hematocrit levels (r = −0.397, *P* = 0.018) (**Table 3**). CA-positive patients also had higher thrombocyte counts than CA-negative patients (*P* = 0.042) (**Figure 1B**), but CA titers did not significantly correlate with thrombocyte counts, albeit significance was borderline (r = 0.328, *P* = 0.054). Furthermore, CA-positive patients had lower monocyte counts when compared to CA-negative patients (*P* = 0.002) (**Table 2** and **Figure 1B**), and CA titers moderately correlated with monocyte counts as well (r = −0.425, *P* = 0.011) (**Table 3**). No significant associations with complete blood count parameters were found when CG-positive and CG-negative patients were compared (**Supplementary Table 3**).

### CA Titers Might Be Subject to Seasonal Variations

We found a moderate correlation between CA titers and mean daily temperatures on the days when blood was drawn (r = 0.456, *P* = 0.006) (**Table 3** and **Supplementary Table 1**). No such associations were found for CG concentrations (**Table 3**).

### CGs, but Not CAs, Are Linked to Basal Serum Tryptase Levels and ColdU Duration

CG concentrations strongly correlated with basal serum tryptase levels (r = 0.733, *P* = 0.025) and ColdU duration (r = 0.683, *P* = 0.042). CA titers were not linked to these features (**Table 3**).

## Discussion

In our study, a sizeable rate of ColdU patients tested positive for CAs and CGs. More importantly, patients who expressed these cold-reactive antibodies differed from those who did not in demographic, clinical and laboratory markers. Our results suggest that CAs and CGs, in a subpopulation of ColdU patients, are linked to the course and possibly the pathogenesis of their disease.

Our ColdU patients showed higher frequencies of positive CA and CG tests than previously reported (16, 36–39). There are several possible explanations for this discrepancy. CA titers in our patients were low (ranged from 1 to 16), and CG concentrations averaged 150 mg/L with a maximum of 189 mg/L. Earlier studies on ColdU did not report CA titers or CG concentrations and may have used higher thresholds for classifying patients as positive or used assays that differ from ours in sensitivity. Importantly, CAs and CGs may be underdiagnosed in ColdU due to improper sample handling (26, 49), and we very likely avoided false negative results by having blood samples drawn at tertiary centers specialized in CA and CG analysis. Other explanations include differences in patient populations and times of sampling. Our study population entailed patients with several different forms of ColdU, and 37% had atypical types of ColdU including systemic atypical ColdU (*n* = 10), localized ColdU (*n* = 1), and localized cold reflex urticaria (*n* = 2). Although we did not detect differences in CAs or CGs between ColdU subtypes (data not shown), possibly due to the low number of patients, such differences may exist. None of the previous ColdU studies report on sampling times, and our study showed a correlation between CA titers and mean daily ambient temperatures on the days when blood for CAs was drawn. CAs, in patients with ColdU, may get depleted in cold seasons due to reactions to cold ambient air. Bendix et al. analyzed CA titers of 276 healthy blood donors in January and July and observed no seasonal variations in CA titers (50). This may suggest that lower CA levels in cold seasons may be linked to depletion of CAs in ColdU.

CA titers in our ColdU were significantly linked to a demographic (i.e., female gender), clinical (i.e., reactivity to cold ambient air, UCT scores), and laboratory features (i.e., lower erythrocyte counts, hemoglobin levels, hematocrit levels, and monocyte counts). Studies in hematology have shown that CA titers <64 do not necessarily indicate that CAs are not pathogenically relevant at 4°C (51). Even fatal autoimmune hemolytic anemia was reported in a patient with a CA titer 16 (52). Agglutination and lysis of erythrocytes are not related directly to the CA titer and rather appear to depend on the density of CA receptors on the erythrocyte surface (23).

Our CA-positive patients were significantly more often female, which is consistent with previous reports (30, 50, 53). Furthermore, higher CA titers were linked to a female gender. Both may be explained by the presence of two X chromosomes, which carry immune response genes, or hormonal factors that influence CA expression (54).

In our study, a positive CA test was associated with a higher rate of reactions triggered by cold ambient air or immersion in cold water and aggravated by increased summer humidity. This suggests that CA-positive patients are more sensitive to cold exposure, and that CAs in the skin might get activated by convective cooling due to moving currents (e.g., immersion in cold water), and by rapid evaporative cooling (e.g., vaporization of water directly from the skin in humid environments).

A positive CA test was also linked to a higher frequency of angioedema triggered by the ingestion of cold foods or beverages. The mechanism remains unexplained. Interestingly, I-antigen was detected in the gastric mucosa of animals (23), and Innet et al. described a CA-positive patient who experienced intermittent anemia and preferred to drink warm liquids (55). The fact that CA-positive patients react to cold ambient air and more severely (angioedema due to cold foods/drinks) may explain our finding that they also have lower disease control.

Our study does not explain why some patients with ColdU have CAs. It is known that infections, for example with *Mycoplasma pneumoniae* or *Streptococcus pneumoniae*, can cause cold agglutinin disease (15, 21, 49, 56, 57). These bacteria display I-related antigens on their surface and can trigger CA production (31), and ColdU has often been linked to preceding infections (1). Up to one third of ColdU patients have been reported to benefit from treatment with doxycycline, and 19% showed full remission (58), but the mechanisms for such improvements have not yet been explained. Furthermore, doxycycline is also effective against *Mycoplasma pneumoniae* (59) and *Streptococcus pneumoniae* (60).

Since our patients’ serum was tested for agglutination of group O erythrocytes, their CAs are likely auto-anti-I (54). Pruzanski and Katz (23) described the biological diversity of CAs almost 40 years ago, which not only agglutinate erythrocytes, but also have complex interactions with other cells expressing the Ii-antigen (i.e., granulocytes, monocytes, macrophages, lymphocytes, thrombocytes, fibroblasts). Interestingly, higher CA titers in our patients correlated with lower erythrocyte and monocyte counts. It is therefore tempting to speculate that CAs in ColdU get activated when the skin is cooled and bind to cells that express the Ii-antigens (like in CA disease), thereby reducing their numbers. These cells may then activate complement and generate C3a and C5a, which are potent mast cell activators (28).

Defined cutoff levels of CGs, which would aid in the interpretation of our results, do not yet exist for ColdU. Our CG-positive patients were referred to a rheumatologist for further evaluation, but none of them received a diagnosis of a rheumatologic disease. Nonetheless, we found that higher CG levels in our ColdU patients were strongly associated with longer ColdU duration and higher basal serum tryptase levels. The latter suggests that CGs may promote mast cell degranulation, as serum tryptase is derived from mast cells and ColdU is a mast-cell driven disease (61). Costanzi and Coltman demonstrated that ColdU activity can be passively transferred to normal recipients with IgG or IgG-IgM CGs obtained from sera of patients with ColdU and cryoglobulinemia (43). All of our CG-positive ColdU patients had the IgG CG-isotype, and 7 of 9 also had the IgM CG-isotype.

Only 6% (n = 2) of studied patients had both, a positive CA and CG test. This suggests that having one type of cold protein does not present a risk for having another. Furthermore, our study showed that CAs and CGs are linked to different sets of markers of ColdU.

The major strengths of our study are that it was performed at a single center allowing good comparability of results, and that we obtained detailed history from all patients. However, it also has limitations: (a) we analyzed a relatively low number of patients, which did not allow for meaningful subgroup analyses, for example of patients with typical *vs*. atypical ColdU, (b) CA and CG tests were each performed in specialized laboratories and not repeated, (c) the thermal amplitude of CAs was not determined, and (d) we did not perform the same laboratory tests in a control group of healthy, age-matched individuals who may have low-titer, low-thermal amplitude CAs and low-concentration CGs in their serum (**Table 1**).

Despite these limitations, this is the first study of CAs and CGs in ColdU to show their association with clinical features, and the fact that such links exist suggests relevance. Our results, therefore, encourage further studies on CAs and CGs in ColdU to explore their function, their use as markers and their impact on treatment responses.

## Data Availability Statement

The original contributions presented in the study are included in the article/**Supplementary Material**. Further inquiries can be directed to the corresponding author.

## Ethics Statement

The studies involving human participants were reviewed and approved by National Medical Ethics Committee of the Republic of Slovenia. The patients/participants provided their written informed consent to participate in this study.

## Author Contributions

MB initiated the study. MB and MK evaluated the patients. All authors substantially contributed to study design, analysis, interpretation of data, and manuscript development. All authors contributed to the article and approved the submitted version.

## Conflict of Interest

MB has been a speaker and an advisor for Novartis. MM is or recently was a speaker and/or advisor for and/or has received research funding from Allakos, Aralez, ArgenX, AstraZeneca, Celldex, Centogene, CSL Behring, FAES, Genentech, GIInnovation, Innate Pharma, Kyowa Kirin, Leo Pharma, Lilly, Menarini, Moxie, MSD, Novartis, Roche, Sanofi/Regeneron, Third HarmonicBio, UCB, and Uriach.

The remaining authors declare that the research was conducted in the absence of any commercial or financial relationships that could be construed as a potential conflict of interest.

## Abbreviations

CA(s), cold agglutinin(s); ColdU, cold urticaria; CG(s), cryoglobulin(s); CR(s), cold-induced reaction(s); CRP, C-reactive protein; CST(s), cold stimulation test(s); CSTT, critical stimulation time threshold; CTT, critical temperature threshold; Ig, immunoglobulin; IgA, immunoglobulin A; IgE, immunoglobulin E; IgG, immunoglobulin G; IgM, immunoglobulin M; IQR, interquartile range; SD, standard deviation; UCT, Urticaria Control Test.
