# Supplementary material for: Cold Agglutinins and Cryoglobulins Associate With Clinical and Laboratory Parameters of Cold Urticaria
Source: Front Immunol. 2021 Apr 29;12:665491. doi: 10.3389/fimmu.2021.665491 (PMC8117240; doi:10.3389/fimmu.2021.665491)
Supplement: Supplementary file 1 [file DataSheet_1.pdf]

Supplementary Material

Supplementary Table 1 Variables linked to cold agglutinins

Patient no.	Sex	CA test[a]	CA titer[b]	UCT score	Hemoglobin level (g/L)	Erythrocyte count (10^{12}/L)	Monocyte count (10^{9}/L)	Thrombocyte count (10^{9}/L)	Mean daily Temp. (°C)[c]
1	F	Neg	0	13	141	4.61	1.01	340	0
2	M	Pos	1	5	137	4.44	0.38	271	17
3	F	Pos	2	3	132	4.51	0.35	468	25
4	M	Neg	0	16	149	4.81	0.57	236	18
5	F	Pos	4	3	146	4.85	0.50	209	17
6	F	Pos	4	7	140	4.24	0.57	330	13
7	M	Neg	0	11	147	4.66	0.61	257	15
8	F	Pos	1	12	138	4.58	0.37	275	21
9	F	Neg	0	6	154	4.80	0.60	266	4
10	M	Neg	0	16	159	4.92	0.91	237	16
11	F	Pos	2	12	146	4.74	0.64	318	21
12	F	Pos	4	14	139	4.32	0.41	203	22
13	M	Neg	0	9	154	5.22	0.50	371	21
14	F	Pos	1	7	130	4.02	0.05	301	18
15	F	Neg	0	6	130	4.19	0.79	272	2
16	M	Pos	2	13	143	4.99	0.71	365	21
17	M	Neg	0	15	150	5.13	0.61	282	6
18	M	Neg	0	16	159	5.43	0.56	259	17
19	F	Neg	0	15	135	4.37	0.69	269	15
20	F	Pos	2	9	134	4.17	0.58	292	13
21	F	Pos	4	11	120	4.27	0.20	274	22
22	F	Neg	0	16	145	4.42	0.69	222	2
23	F	Pos	16	12	145	5.06	0.37	335	10
24	F	Neg	0	14	151	4.73	0.53	170	2
25	F	Neg	0	10	146	4.76	0.38	197	10
26	F	Neg	0	16	142	4.43	0.53	220	13
27	M	Neg	0	16	162	6.02	0.53	150	8
28	F	Pos	1	12	134	4.69	0.40	285	0
29	F	Pos	2	7	123	4.44	0.33	248	10
30	M	Neg	0	13	146	4.98	0.35	208	13
31	F	Pos	2	10	128	3.98	0.33	228	13
32	M	Neg	0	13	149	4.90	0.41	222	7
33	M	Neg	0	5	161	5.27	0.47	271	5
34	F	Pos	1	11	137	4.22	0.30	222	7
35	F	Neg	0	4	139	4.69	0.52	233	2

[a]Cold agglutinin test performed at 4°C. [b]The numbers are inverse of the maximum serum dilution at which agglutination can be seen (i.e., CA titer 4 = agglutination at 1:4 dilution). [c]Mean daily temperature on the day when blood was drawn was obtained from the national weather service website (http://meteo.arso.gov.si/met/en/). *Abbreviations*: CA, cold agglutinin; F, female; M, male; Neg, negative; Pos, positive; Temp., temperature; UCT, Urticaria Control Test.

Supplementary Table 2 Variables linked to cryoglobulins

Patient no.	CG test	CG conc. (mg/L)	CG Ig isotype(s)	ColdU duration (months)	Lymphocyte count (10^9/L)	Basal serum tryptase level (ng/mL)
1	Pos	137	IgG, IgM, IgA	70	2.13	3.66
2	Neg	–	–	14	1.55	3.24
3	Neg	–	–	24	2.58	3.41
4	Neg	–	–	60	2.49	11.60
5	Neg	–	–	336	1.39	8.15
6	Neg	–	–	9	1.22	15.90
7	Neg	–	–	4	1.94	5.34
8	Neg	–	–	50	1.63	5.32
9	Pos	189	IgG	169	2.89	7.57
10	Neg	–	–	120	1.52	6.19
11	Neg	–	–	228	2.49	23.00
12	Neg	–	–	156	2.68	3.57
13	Neg	–	–	60	2.44	ND
14	Neg	–	–	99	1.94	5.35
15	Pos	124	IgG, IgM	24	1.88	3.83
16	Neg	–	–	24	1.40	5.50
17	Neg	–	–	240	1.99	7.79
18	Neg	–	–	10	1.34	4.44
19	Pos	184	IgG, IgM	252	2.67	13.60
20	Neg	–	–	276	1.70	5.49
21	Neg	–	–	72	1.20	3.96
22	Neg	–	–	120	2.61	6.37
23	Pos	153	IgG, IgM, IgA	216	2.62	5.55
24	Neg	–	–	384	1.80	7.41
25	Neg	–	–	2	1.13	6.00
26	Neg	–	–	22	2.06	4.55
27	Neg	–	–	15	2.01	12.70
28	Pos	187	IgG, IgM	84	1.72	6.09
29	Neg	–	–	8	1.75	3.05
30	Pos	106	IgG, IgM	60	1.22	2.92
31	Neg	–	–	42	1.95	5.10
32	ND	–	–	8	1.47	2.90
33	Pos	138	IgG, IgM	72	1.49	6.06
34	ND	–	–	22	2.17	5.33
35	Pos	150	IgG	11	2.12	14.00

Abbreviations: conc., concentration; CG(s), cryoglobulin(s); CRP, C-reactive protein; Ig, immunoglobulin; IgA, immunoglobulin A; IgG, immunoglobulin G; IgM, immunoglobulin M; Neg, negative; ND, not done/determined; Pos, positive.

Supplementary Table 3 Characteristics of patients with a positive vs. negative cryoglobulin test

**Parameter**	**CG test**			
	**Total** **N = 33**	**Negative** **N = 24**	**Positive** **N = 9**	***P* value (positive vs. negative)**
Female sex	22 (67)	15 (63)	7 (78)	0.681
Triggers				
Cold ambient air	27 (82)	20 (83)	7 (78)	1.000
Immersion in < 25°C water	18 (55)	13 (54)	5 (56)	0.627
Higher summer humidity levels	15 (46)	11 (46)	4 (44)	1.000
Ingestion of cold foods/drinks	18 (55)	14 (58)	4 (44)	0.697
UCT score	12 (7−15)	12 (8−16)	12 (6−13)	0.310
CST results				
Positive ice cube test	20 (61)	16 (67)	4 (44)	0.425
Positive Temp*Test*®	12 (36)	11 (46)	1 (11)	0.107
CSTT (s)	240 (30−300); N = 18	150 (30−300); N = 14	300 (165−300); N = 4	0.182
CTT (°C)	19 (15−25); N = 12	21 (16−25); N = 11	14; N = 1	0.242
Laboratory findings				
Erythrocyte count (10^{12}/L)	4.7 ± 0.4	4.7 ± 0.5	4.7 ± 0.3	0.685
Hemoglobin concentration (g/L)	142.6 ± 10.6	142.5 ± 11.0	142.8 ± 10.0	0.948
Hematocrit level (%)	41.7 ± 3.4	41.6 ± 3.4	41.9 ± 3.5	0.828
Thrombocyte count (10^9/L)	268.6 ± 63.2	266.0 ± 70.1	275.4 ± 42.3	0.708
Monocyte count (10^9/L)	0.5 ± 0.2	0.5 ± 0.2	0.6 ± 0.2	0.266
Leukocyte count (10^9/L)	7.0 ± 1.6	6.8 ± 1.5	7.5 ± 2.0	0.269
Neutrophil count (10^9/L)	4.3 ± 1.2	4.2 ± 1.2	4.4 ± 1.5	0.639
Lymphocyte count (10^9/L)	1.9 ± 0.5	1.9 ± 0.5	2.1 ± 0.6	0.285
Eosinophil count (10^9/L)	0.2 ± 0.3	0.2 ± 0.1	0.3 ± 0.4	0.329
Basophil count (10^9/L)	0.04 ± 0.03	0.04 ± 0.03	0.05 ± 0.02	0.094
CRP (mg/L)	1.7 (0.5−3.5)	1.5 (0.6−3.5)	2.0 (0.5−4.8)	0.984
Basal serum tryptase (ng/mL)	5.5 (4.1−7.7); N = 32	5.5 (4.4−7.8); N = 23	6.1 (3.7−10.6)	0.850
Total IgE (IU/mL)	113 (47−232); N = 29	94 (30−232); N = 21	143 (62−1101); N = 8	0.341
Mean daily Temp. on the day when blood was drawn	11.1 ± 7.6	12.4 ± 6.6	6.9 ± 9.4	0.077

Data are given as no. (%), mean ± SD, and median (IQR). If data was not obtained in all patients, patient numbers are displayed as "*N*" next to results. Statistical significance of differences between patient groups was calculated by Fisher's Exact test, Mann-Whitney U test, and Independent-samples T test. No statistically significant *P* values were found. *Abbreviations*: CG, cryoglobulin; ColdU, cold urticaria; CR(s), cold-induced reaction(s); CRP, C-reactive protein; CST, cold stimulation test; CSTT, critical stimulation time threshold; CTT, critical temperature threshold; IQR, interquartile range; *N*, number of patients; s, second; SD, standard deviation; UCT, Urticaria Control Test.
